# The Effects of Hormones and Vaginal Microflora on the Glycome of the Female Genital Tract: Cervical-Vaginal Fluid

**DOI:** 10.1371/journal.pone.0158687

**Published:** 2016-07-20

**Authors:** Bernard J. Moncla, Catherine A. Chappell, Brian M. Debo, Leslie A. Meyn

**Affiliations:** 1 Department of Obstetrics, Gynecology and Reproductive Sciences, University of Pittsburgh, Pittsburgh, Pennsylvania, United States of America; 2 Magee-Womens Research Institute, Pittsburgh, Pennsylvania, United States of America; 3 Department of Cell and Developmental Biology, University of Pennsylvania, Philadelphia, Pennsylvania, United States of America; Inserm; Univ. Lille; CHU Lille, FRANCE

## Abstract

In this study, we characterized the glycome of cervical-vaginal fluid, collected with a Catamenial cup. We quantified: glycosidase levels; sialic acid and high mannose specific lectin binding; mucins, MUC1, MUC4, MUC5AC, MUC7; and albumin in the samples collected. These data were analyzed in the context of hormonal status (day of menstrual cycle, hormonal contraception use) and role, if any, of the type of the vaginal microflora present. When the Nugent score was used to stratify the subjects by microflora as normal, intermediate, or bacterial vaginosis, several important differences were observed. The activities of four of six glycosidases in the samples from women with bacterial vaginosis were significantly increased when compared to normal or intermediate women: sialidase, *P* = <0.001; α-galactosidase, *P* = 0.006; β-galactosidase, *P* = 0.005; α-glucosidase, *P* = 0.056. Sialic acid binding sites as measured by two lectins, *Maackia amurensis* and *Sambucus nigra* binding, were significantly lower in women with BV compared to women with normal and intermediate scores (*P* = <0.0001 and 0.008 respectively). High mannose binding sites, a measure of innate immunity were also significantly lower in women with BV (*P* = <0.001). Additionally, we observed significant increases in MUC1, MUC4, MUC5AC, and MUC7 concentrations in women with BV (*P* = <0.001, 0.001, <0.001, 0.02 respectively). Among normal women we found that the membrane bound mucin MUC4 and the secreted MUC5AC were decreased in postmenopausal women (*P* = 0.02 and 0.07 respectively), while MUC7 (secreted) was decreased in women using levonorgestrel-containing IUDs (*P* = 0.02). The number of sialic acid binding sites was lower in the postmenopausal group *(P* = 0.04), but the number of high mannose binding sites, measured with Griffithsin, was not significantly different among the 6 hormonal groups. The glycosidase levels in the cervical-vaginal mucus were rather low in the groups, with exception of α-glucosidase activity that was much lower in the postmenopausal group (*P*<0.001). These studies present compelling evidence that the vaginal ecosystem responds to the presence of different vaginal microorganisms. These effects were so influential that it required us to remove subjects with BV for data interpretation of the impact of hormones. We also suggest that certain changes occurring in vaginal/cervical proteins are due to bacteria or their products. Therefore, the quantitation of vaginal mucins and lectin binding offers a new method to monitor bacteria-host interactions in the female reproductive tract. The data suggest that some of the changes in these components are the result of host processing, such as the increases in mucin content, while the microflora is responsible for the increases in glycosidases and the decreases in lectin binding. The methods should be considered a valid marker for insult to the female genital tract.

## Introduction

Secreted cervical mucus coats the vaginal epithelium and cervix, forming a protective physical and biochemical barrier. The mucus is composed of 2% to 5% mucin glycoproteins and 1% other secreted proteins, including antibodies, antibacterial proteins, and peptides. Secreted mucins, the gel-forming component of mucus, are large, highly glycosylated proteins (10–40 MDa) that form a viscoelastic gel [1–6]. Branched carbohydrate chains (3–10 sugars in length) consisting of N-acetyl-glucosamine, N-acetyl-galactosamine, galactose, fucose, and sialic acid account for up to 80% of the weight of mucins [7, 8].

Glycosidic and proteolytic degradation of gastrointestinal mucus by enteric bacteria is well documented and has been linked to the pathogenesis of inflammatory conditions in the gastrointestinal tract [9, 10].

Mucins and the viscoelastic nature of mucus gels protect epithelial surfaces by providing lubrication, by creating an unstirred layer through which pathogens must pass, and by physically trapping pathogens. There are two different general types of mucins, gel-forming mucins and membrane bound mucins. The functions of these proteins are diverse and complex. Examples of their roles in biology include: cell adhesion and bacterial attachment; cellular differentiation; and the pathogenesis of cancer [11–14]. One of the most important characteristics of mucins and other glyco-molecules is the presence of sialic acid. There about 50 different types of sialic acids (SA). Both the type of sialic acid and its linkage to subordinate sugars are important in the determination of how the SA functions [15, 16]. One of the best examples is the hemagglutinin proteins of the Influenza A viruses. The human Influenza A virus targets sialic acid in the α 2-6Gal(NAc) configuration while the avian Influenza hemagglutinin binds sialic acid in the α 2-3Galβ-1 configuration. If the avian virus were to mutate so it recognized the α 2-6Gal(NAc) configuration, it could jump species and it may start infecting humans [17]. It is hemagglutinin specificity that defines the tissue to which the viruses may attach and subsequently infect, for review see [15, 16].

Upper respiratory tract mucosal secretions are known to trap many microorganisms, which aids in their removal [18]. Cervical mucus also traps pathogens as small as herpes simplex virus, suggesting that cervical mucus may also play a critical role in the innate immunity of the female reproductive tract [19–21]. Conversely, some pathogens such as influenza virus produce sialidases that alter the local environment by exposing the sub-terminal galactose or N-acetyl galactosamine that facilitates colonization by opportunistic bacteria [15, 16].

We have evaluated vaginal antimicrobial peptides, pH, and viscosity as they relate to innate immunity and the use of microbicides. Chappell et al., using the same group of women used in this report, found significant differences in pH, viscosity and protein content were associated with hormonal status or type of hormonal contraceptive used [22]. A subsequent report detailed the studies of innate anti-HIV, HSV-1 and HSV-2 activity among women with different hormonal status and found anti-HIV activity was significantly lower in post-menopausal women [22, 23]. The lower innate immunity we observed in post-menopausal women was correlated to lower protein content in the CVF of the post-menopausal group. Post-menopausal women samples had about 40% less protein than all of the reproductive age women combined (*P*<0.001) [22, 23].

Genetic studies have identified about 20 genes for mucin production, but the heterogeneity observed when mucins are purified suggests that many post-production modifications occur such as the addition of carbohydrate side chains [24, 25]. The glycoproteins’ sugar side-chains may function as lectin-like receptors for immunological events, modulate microbial flora and vaginal fluid viscosity, and vary with estrogen levels and allow for survival and migration of spermatozoa [1, 5, 6, 26]. We have previously reported on the cervical vaginal lavages (CVLs) of our cohort of women and also observed decreased viscosity among the hormonal groups in addition to changes in the sugar side-chains detected by lectin binding, but the impact of the hormonal status was much less than that of the vaginal microflora [22, 23, 27, 28].

The carbohydrate side chains protect the protein core from proteolytic attack but are subject to cleavage by glycosidases. In the vagina, endogenous microflora produce glycosidases and sulfatases that slowly alter the mucus proteins; this leads to their eventual turnover [20, 29]. Many times the carbohydrate chain terminates in a sialic acid molecule that carries a negative charge. This charge is important in determining the function of the molecule and what message it will send. It also is important for the tertiary structure of many different types of molecules, for review see [12, 16, 30, 31]. Intra-vaginal products may affect the protective mucus gel directly or indirectly. Some may directly absorb the mucus gel proteins and carry them away or alter the endogenous enzymes to increase or decrease their respective activities in the vagina, thus changing the delicate equilibrium and altering glycoprotein structure and function (unpublished observations, [22, 23, 27, 28]).

We know that women with bacterial vaginosis are at increased risk of HIV acquisition and low birth weight babies [32–34]. The vaginal microbiome shifts from a normal aerobic flora consisting of gram positive rods and cocci to a flora of women with BV that consists primarily of anaerobic gram negative rods and cocci [34]. We have already reported the changes observed in the vaginal glycome and demonstrated that some of these changes are related to the presence and increase of bacterial enzymes present in the CVL [22, 23, 27, 28].

We are developing intra-vaginal products for the prevention of HIV transmission. One of the parameters is the composition of the mucus layer of the female reproductive tract. However, there are several factors that confound these studies before a baseline can be established. It was also quite likely our samples could be affected by the stage of the menstrual cycle, the type of birth control used, or type of microbiota present [1, 6, 35]. We set out to characterize the protein structures of the cervical-vaginal proteins and to measure the impact of hormonal status and vaginal microbiota on these proteins. We have previously shown the changes in vaginal enzymes and their correlation to changes in lectin binding to the proteins in CVL [27, 28].

Bacterial enzymes, which degrade mucins and other glycoproteins, may play an important function by altering the glycoproteins/mucins, which in turn may impact their turnover intra-vaginally. Many of these enzymes are also known to be associated with bacterial vaginosis and low birth weight babies [7, 36–43]. Others have reported differences in composition of the various samples taken from different locations in the lower female genital tract [44] as well as differences in sampling methods [45, 46]. In this study we report the changes in membrane bound (MUC1 and MUC4) and gel-forming mucins (MUC5AC and MUC7) relative to the influence of hormones and the presence of the abnormal vaginal flora of bacterial vaginosis.

## Material and Methods

### Study Population

This was a secondary analysis of samples collected as part of a study of the physical properties of vaginal fluid, and the complete methods describing the study populations are described elsewhere [22]. Written informed consent was obtained following a protocol approved by the University of Pittsburgh IRB. Women were excluded if they were: breastfeeding or pregnant; presented vaginal symptoms; had a hysterectomy; had been diagnosed with any cervical or vaginal infections or had used any antimicrobials in the past 14 days; had used any vaginal devices or vaginally-applied products (excluding tampons) in the past week. Upon enrollment the women had: an OraQuick advance rapid HIV test; a pregnancy test; their demographic information recorded; height and weight taken and medical, gynecologic and sexual histories taken. Cervicovaginal fluid(CVF) was collected from 165 women and characterized as: post-menopausal; first 14 days of cycle, (1–14 days of menstrual cycle); second 14 days of cycle, (15–30 days of menstrual cycle); oral contraceptives; depo- medroxyprogesterone acetate (DMPA); or women using the Mirena intrauterine device (IUD). Vaginal smears were Gram stained and evaluated using the Nugent criteria [34].

### Sample Collection

Cervical-vaginal fluid (CVF) was collect by inserting an Instead Softcup^®^ to the base of cervix. The fluid is allowed to collect for 45 min in place and the cup removed and placed into a 50 mL conical centrifuge tube and stored at 4°C until processing. The samples were transported to the laboratory within one hour for processing.

### CVF processing

The CVF samples were collected by centrifuging the cups at 2,000 x g for 10 minutes and the protein laden material scraped out and the volumes estimated. These samples were stored at -80°C.

### ELISA and ELLA assays

We used the same methodology for both the enzyme-linked immunosorbent assay (ELISA, used to measure mucins and human serum albumin) and the enzyme-linked lectin assay (ELLA, used to measure the carbohydrate structures). CVFs were diluted to give a final SDS (Sigma-Aldrich, St. Louis MO) concentration of 1% in 50mM sodium carbonate buffer. The samples were heated in a boiling water bath for 5 min, added to flat bottom 96 well clear microtiter plates (Nunc, Thermo Fisher, 75, Panorama Creek Dr., Rochester, NY 14625), at a concentration of 300–500 ng protein /100μL and allowed to air dry in an oven set to 40°C overnight. Plates were washed 4 times in phosphate buffered saline (PBS), blocking buffer was added (PBS plus 0.5% polyvinyl alcohol 30–50 KDa (Sigma-Aldrich, St. Louis, MO) and incubated at room temperature for 1 hr. Plates were washed 4 X with PBS. Horse radish peroxidase labeled lectins or antibodies (Vector Laboratories, Burlingame, CA 94010 Abcam, Cambridge, MA 02139, Novus Biologicals, Litton CO 80120) were diluted to the optimum concentration in PBS containing 0.5% PVA and 0.05% Tween 20 and 50 μL was added to each well. Plates were covered with aluminum foil and incubated on an orbital shaker at 300 rpm for 1 hour. Plates were washed 4 X with 0.05% Tween 20 in PBS. The plates were developed using 3,3′,5,5′-Tetramethylbenzidine (TMB) Liquid Substrate System for ELISA (Sigma-Aldrich, St. Louis, MO, 63178) and incubated for an appropriate time. The reactions were stopped by the addition of 1N H_2_SO_4_ and the optical densities read with a DTX880 Multimode Detector (Beckman-Colter, Fullerton, CA 92834–3100 USA). Each plate included dilutions of MCF7 cell lysate (Santa Cruz Inc. Santa Cruz, CA.) as a mucin standard and an appropriate dilution of cervical mucus that was derived from a pool of four different women (Lee Bio-Solutions, St. Louis, MO). ELISA data are presented as ng/ng protein. Griffithsin binding was performed as the other lectins except the HIV glycoprotein gp120 (Protein Sciences Corp. Meriden, CT 06450) was used to generate the standard curve. Griffithsin and anti-Griffithsin antibodies were a generous gift from Ken Palmer (University of Louisville, College of Medicine, Louisville, Kentucky).

### Glycosidase assays

Glycosidase assays were performed exactly as previously described, in triplicate, using a modification of the methods in Moncla et al. [15,39]. Briefly, 4-methyllumbelliferyl- sugar derivatives (Sigma-Aldrich, St. Louis, MO) (-α-D-glucoside, β-D-glucoside, α-L-fucoside, β-D-fucoside, α-D-galactoside, β-D-galactoside, and α-D-N-acetyl neuraminic acid) were suspended in ultra-pure water Milli-Q (Millipore, Billerica, MA) to a final concentration of 0.2 mM. Cervical vaginal fluid was diluted to 2mg protein per ml saline and 80 μL placed into the bottom of a glass tube. The samples then received 10 μL of 0.1 M sodium acetate buffer, pH 5.0 and incubated at 37 C in a reciprocating shaker for ten minutes. Reactions were initiated by the addition of 10 μL of the substrate. The reactions were stopped, at appropriate intervals, (from 20 to 60 min) by the addition of 300 μL 0.1M borate buffer pH 9.2 prepared fresh daily. Precipitates were removed by centrifugation at 10,000 x g for 5 min and 100 μL of the clear supernatants transferred to microtiter plate and read in a DTX880 Multimode Detector (Beckman-Coulter, Fullerton, CA), excitation light 365nm emission 450nm.

### Protein concentrations

Protein concentrations were determined using a modification of the Lowry assay [47, 48].

## Results and Discussion

A total of 165 women were enrolled in our study; of these, two samples were lost and not fully analyzed. The samples used are the same samples as used in previous reports [22, 23, 27, 28] and the population’s characteristics are given in Chappell et al. [22].

We estimated the volume of CVF recovered (0.6–0.8 mL/participant) and the average protein concentration of the all samples was 1.4 mg/mL. We analyzed the albumin content of the entire sample since we believed it should remain constant and it did, see Tables 1 and 2.

**Table 1:** Summary of enzyme activity, lectin binding and mucin content of women stratified by microflora type, Nugent criteria.

CVF	Nugent Score			*P*-value^3^
	Normal (n = 89)	Intermediate (n = 23)	Bacterial Vaginosis (n = 23)	
Glycosidases^1^				
Sialidase	0 (0–0.14)^2^	0 (0–0.17)	0.08 (0–0.25)	<0.001
α-fucosidase	0.007 (0–0.75)	0.005 (0–0.02)	0.006 (0–0.17)	0.51
α-galactosidase	0 (0–0.16)	0 (0–0.04)	0.004 (0–0.07)	0.006
β-galactosidase	0 (0–0.43)	0 (0–0.03)	0.003 (0–0.03)	0.005
α-glucosidase	0.12 (0–12.86)	0.16 (0–0.71)	0.28 (0–1.16)	0.056
β-glucosidase	0 (0–0.07)	0 (0–0.02)	0 (0–0.01)	0.23
Lectin binding				
*Maackia amurensis*	0.20 (0.06–0.60)	0.17 (0.07–0.50)	0.12 (0.07–0.26)	<0.001
*Sambucus nigra*	0.15 (0.06–1.03)	0.18 (0.09–0.85)	0.12 (0.07–0.29)	0.008
Griffithsin	186.32 (14.98–500)	171.29 (9.76–500)	69.13 (9.87–247.32)	<0.001
HSA	0.07 (0.05–0.10)	0.07 (0.06–0.09)	0.07 (0.06–0.10)	0.25
Mucins				
MUC1	0.35 (0.09–4.77)	0.42 (0.20–1.70)	1.13 (0.19–45.68)	<0.001
MUC4	0.49 (0.11–1.32)	0.53 (0.25–1.72)	0.72 (0.31–2.12)	0.001
MUC5AC	0.22 (0.10–0.49)	0.25 (0.15–0.40)	0.30 (0.17–0.60)	<0.001
MUC7	0.17 (0.04–0.70)	0.18 (0.06–0.44)	0.23 (0.06–0.71)	0.02

^1^Enzyme activity is expressed as μM hydrolyzed (4MU-glycosides) per min per mg of protein at pH 5.0. SNA and MAL binding is expressed as ng lectin bound per ng protein. Mucin content is reported as μg mucin per μg protein. Griffithsin binding is in pg bound/ng protein.

^2^Data presented as median (range)

^3^*P*-value from Kruskal-Wallis

**Table 2:** Summary of enzyme activity, lectin binding and mucin content of women stratified by hormonal status.

CVF^1^	Postmenopausal	Premenopausal, Proliferative	Premenopausal, Follicular	OCP	DMPA	IUD	*P*^3^
	(n = 23)	(n = 19)	(n = 23)	(n = 24)	(n = 19)	(n = 25)	
Glycosidases^2^							
• Sialidase	0 (0–2.44)	0 (0–6.21)	0 (0–6.90)	0 (0–0.07)	0 (0–0.22)	0 (0–1.16)	0.08
• α-fucosidase	0.006 (0–0.02)	0.008 (0–0.46)	0.006 (0–0.02)	0.006 (0–0.01)	0.008 (0–0.75)	0.007 (0–0.05)	0.4
• α-galactosidase	0 (0–0.05)	0.001 (0–0.16)	0 (0–0.09)	0 (0–0.10)	0 (0–0.10)	0 (0–0.05)	0.58
• β-galactosidase	0 (0–0.15)	0.001 (0–0.16)	0 (0–0.43)	0 (0–0.08)	0 (0–0.09)	0 (0–0.05)	0.6
• α-glucosidase	0.005 (0–1.51)	0.15 (0.001–12.86)	0.11 (0–0.68)	0.15 (0–0.69)	0.19 (0–5.96)	0.10 (0–0.71)	<0.001
• β-glucosidase	0 (0–0.10)	0 (0–0.05)	0 (0–0.07)	0 (0–0.003)	0 (0–0.05)	0 (0–0.02)	0.39
HSA	0.07 (0.05–0.09)	0.08 (0.06–0.10)	0.08 (0.06–0.10)	0.07 (0.05–0.09)	0.06 (0.05–0.08)	0.07 (0.06–0.09)	0.03
Lectin binding							
*Maackia amurensis*	0.15 (0.07–0.37)	0.24 (0.07–0.54)	0.18 (0.06–0.60)	0.19 (0.09–0.35)	0.21 (0.10–0.53)	0.17 (0.08–0.45)	0.045
*Sambucus nigra*	0.21 (0.06–0.54)	0.19 (0.07–1.03)	0.15 (0.06–0.75)	0.13 (0.09–0.33)	0.20 (0.11–0.85)	0.15 (0.09–0.51)	0.043
Griffithsin	155.26 (14.20–500)	188.71 (18.99–500)	195.06 (9.76–500)	156.49 (23.76–500)	217.46 (43.62–500)	200.07 (19.11–285.09)	0.28
Mucins							
MUC1,	0.29 (0.05–0.98)	0.44 (0.13–1.70)	0.35 (0.13–3.75)	0.34 (0.14–4.77)	0.43 (0.22–4.21)	0.33 (0.09–1.07)	0.26
MUC4,	0.32 (0.33–1.83)	0.52 (0.29–1.72)	0.44 (0.25–1.32)	0.50 (0.11–0.76)	0.48 (0.31–1.25)	0.50 (0.24–0.72)	0.02
MUC5AC	0.19 (0.07–0.29)	0.25 (0.16–0.49)	0.23 (0.15–0.43)	0.22 (0.10–0.40)	0.27 (0.15–0.40)	0.20 (0.10–0.43)	0.07
MUC7	0.24 (0.09–0.69)	0.19 (0.04–0.42)	0.18 (0.06–0.70)	0.17 (0.06–0.53)	0.18 (0.07–0.44)	0.13 (0.04–0.24)	0.02

^1^Data from women with BV have been omitted from these analyses.

^2^Data presented as median (range), enzyme activity, μM hydrolyzed per min per mg of protein; SNA and MAL binding is expressed as ng lectin bound per ng protein. Mucin bound is reported as μg bound per μg protein. Griffithsin binding is in pg bound/ng protein.

^*3*^*P*-value from Kruskal-Wallis test

### Impact of microbial flora

The Nugent criteria were used to characterize the vaginal microflora as *Lactobacillus*-predominant, intermediate or consistent with BV. Post-menopausal women are excluded from these analyses because the Nugent score has only been validated for use in women of reproductive age. When we stratified the data by glycosidases and lectin binding in the CVF across the Nugent categories, we observed differences in both glycosidase activities and glycan patterns.

These changes are significantly associated with the changing bacterial composition (Table 1). We have recently demonstrated that these changes include the increases in bacterial glycosidases that are concurrent with conversion of the vaginal microflora from normal to abnormal. The glycosidases have long been studied but it is only recently that their impact on the vaginal proteins has been demonstrated. The changing microflora is also correlated with increases in certain mucins and changes in the carbohydrate structures of the vaginal proteins [27, 28].

### Impact on glycosidase

The levels of glycosidases, in specific activity, in the CVFs of normal and intermediate women were very low; however, there were statistically significant increases in: sialidase, α-galactosidase, β-galactosidase and α-glucosidase (*P* = <0.001, 0.006, 0.005 and 0.056 respectively), but not α-fucosidase or β-glucosidase in women with BV, see Table 1. We saw the same enzymes increasing in the CVLs from these women with BV, but the median enzyme values for the CVLs were much higher and ranges were greater [27]. We believe most enzymes appear to be of bacterial origin. Bacteria are seldom observed in cervical mucus Gram stains, but CVLs usually contain >10^9^ bacteria per mL [49]. It is therefore highly likely that the higher enzymatic activity observed in CVFs is of bacterial origin. Further, the activity of the CVF could be the result of contamination upon removal of the Catamenial cup. Attempts to remove the bacteria and yeast from the samples have been unfruitful as the mucins sediment with the bacteria and yeast. We estimate that as much as one third of the CVL protein may be bacterial. Even this amount of bacterial protein should not affect the lectin binding assays since the quantities of sialic acid bearing bacteria would be a small part of the whole; however, bacterial enzymatic activities probably represent most or all of the enzyme activities present. These glycosidases are sufficient to alter the endogenous glycoproteins or cause changes in the glycosylation patterns or both.

### Impact of flora on lectin binding

We observed increased sialidase and glycosidases activities in the women with BV and hypothesized that we would observe significantly lower quantity of α-2,6 and α-2,3 linked sialic acids in these women with BV. Comparing the lectin binding among the women stratified by microflora type, we found a significant decrease in the binding of three lectins, *Maackia amurensis* (MAL, preferentially binds to α-2,3 linked sialic acid, *P* = <0.001), *Sambucus nigra* (SNA, preferentially binds to’α-2,6 linked sialic acid, *P* = 0.008) and Griffithsin (GRFT, binds high mannose structures, *P* = <0.001) among women with BV compared to normal or intermediate women (Table 1). Although 4-methylumbeliferone derivatives assays are quick and easy for the detection of glycosidase enzymes, they do not provide information about the specificity of the enzyme. However, others working with vaginal swabs have demonstrated a sequential carbohydrate hydrolysis of glycoconjugates including SIgG and IgG [50, 51].

The reduction of the sialic acid binding sites in BV is consistent with the association of increased sialidase activity that has long been associated with the syndrome [39, 52, 53]. The correlation of sialidase activity with reduction in SNA and MAL binding supports our earlier observations, demonstrating that sialidase activity is inversely correlated with the number of sialic acid binding sites [27]. The decreases in sialic acid specific lectin binding suggest that the sialidases are active against the glycoproteins in the female reproductive tract. By extension, the other enzymes detected and associated with BV are most likely as active against the glycoproteins as the sialidase. We have previously reported an increased level of two of the epitopes that would likely result from sialic acid removal, terminal β- galactosides and β- N-acetylgalactosides [28]. There are many potential contributors to the enzymatic milieu of the vagina, each of which shape the environment in its own way; for example, two bacteria associated with BV are *Gardnerella vaginalis* and *Prevotella bivia*. Sialidase is produced by all the *P*. *bivia* isolates but only one in four *G*. *vaginalis* isolates [40, 42, 54]. The *Prevotella* sialidase is produced by all strains tested and most active against α-2,6-linked sialic acid; it is cell bound while the *Gardnerella* enzyme is only produced by 1 of 4 isolates and most active against α-2,3-linked sialic acids. *G*. *vaginalis* also excretes large amounts of sialidase into the environment [27]. The two organisms have the same enzyme but given the differences between the two, we would expect them to have very different effects in modeling the vaginal environment. Secreted sialidases have the capability of reaching locations distal to the organisms themselves and in doing so, may remove sialic acid residues from carbohydrate chains far from the organism. This would not only make sialic acids available to other organisms capable of catabolizing them but would open the carbohydrate chains making them vulnerable to exo- and endo- glycosidase attack. Having many different organisms secreting glycosidases with different specificities is advantageous to the microbiome as a whole, not only for nutritional reasons or in invasive processes but also because the necessary genes may be distributed among different members of the population. The sugar moieties may come in many different forms, for example, sialic acids are often linked in an α-2-3, 2–4 or 2–8 linkage to another sugar. Accordingly, many different sialidases are specific for one or more of these linkages. When we use some synthetic substrates for the measurements of glycosidases, we cannot directly observe the subtle nuances that are occurring.

Thus, there are many factors controlling the very complex lectin-glycan interactions. Work with *Streptococcus* spp. and other organisms that have exoglycosidases with exquisite substrate specificities and have shown that these enzymes are important in both promoting colonization and infection [55–57]. King et al. elegantly demonstrated that there is a sequential hydrolytic attack on the sugars on human glycoproteins by exoglycosidases produced by streptococci [56, 58–60]. Thus, it is important to realize that many of these glycosidases must act in concert and in some cases in sequence to effect carbohydrate removal.

The reduction in binding of all three lectins in women with BV was comparable in magnitude, suggesting the underlying mechanism may be the same. There is an impressive reduction of the high mannose present in the cervical mucus of women with BV. Since the high mannose binding is part of the innate immune system, this would lower women’s defenses against pathogens that utilize such sugar motifs such as HIV and HSV [61, 62]. This is one possible explanation of why women with BV have an increased risk of HIV acquisition.

### Impact of flora on MUCs

When we stratified the data according to the Nugent score we omitted the postmenopausal women because the Nugent criterion is not accurate for these women. We found the median values for MUC1, MUC4, MUC5AC and MUC7 were significantly higher in women with BV (N = 23) than women with normal or intermediate Nugent scores (N = 112) (*P* values = <0.001, 0.001, <0.001 and 0.02 respectively), see Table 1. These values are consistent with our unpublished data of the levels of mucins in cervical-vaginal lavage.

These data indicate that the MUC proteins’ synthesis is increased in women with BV. In contrast, we believe the data for the changes in the enzyme levels are due to the changes in the microbiota that results in a milieu rich in enzymes not found in the normal healthy vagina. These enzymes remove MAL, SNA and possible GRFT binding sites. However, the mucins are derived from the cervix. This increase in mucin production is supported by the *in vitro* work that demonstrates that bacterial components stimulate increased mucin production [63, 64]. In BV, the microflora shifts from a *Lactobacillus* dominant type flora to one where the gram negative anaerobic bacteria dominate.

It would seem easy to visualize the predominantly anaerobic gram negative organism associated with BV releasing lipopolysaccharides and other cellular components that would activate cytokines and so on to increase mucin production [63, 65–68]. Mucins also function as attachment sites and decoys for binding. Bacteria may attach to a specific sugar or sugar motifs that are part of the mucins; for example, *Helicobacter pylori* binds to the sialyl-lewis antigen of MUC7, as do *Pseudomonas aeruginosa* and *Haemophilus influenzae* [66, 69–71]. The increased quantities of mucin we measured may be the result of increased biosynthesis of these proteins or an increased rate of shedding, the purpose of which is unknown, but it may be to present the flora of BV with binding site decoys or to release already bound organisms [66].

Cytokines have been used to monitor microbicide safety as indicators of toxicity [72–75]. They are small glycoproteins and may be difficult to measure. There is considerable evidence that pro-inflammatory cytokines up-regulate the mRNAs of some mucins [67, 76]. Because the interactions of the many cytokines *in vivo* are so complicated, we decided to quantify the changes in the some of the end products of the pro-inflammatory cascade, for example the mucins, rather than the cytokines themselves [77, 78].

Our results suggest there is communication between the vaginal microflora and the mucin synthesizing apparatus of the upper female genital tract. Our data support the notion that as the microflora shifts from healthy to abnormal the message shifts to one that results in the increased production of mucin proteins. Conversely, a message could be from the *Lactobacillus* maintaining a normal healthy ecosystem.

After the mucus matures on the surface of the cervix it is shed and descends into the lower reproductive tract where it mixes with vaginal fluids, bacteria and so on. The material from the cervix is produced at a rate of 0.5 to 1 ml per hour. During its residency in the vagina, bacterial and any endogenous enzymes would result in hydrolysis of mucus components. When we lavage the women we should see a mixture of the altered mucus in the vagina and unadulterated cervical mucus that comes directly from the cervix.

### Impact of microflora on evaluation of hormonal status

The data for the effects of the microflora on the mucins tested compelled us to re-evaluate the data stratified by hormonal status without the effect of BV. Those results are presented here. Postmenopausal women had significantly less MUC4 than the women of reproductive age, Table 2 (*P* = 0.02). Among all the women of reproductive age, women using IUDs had lower levels of MUC7 in the CVF (*P* = 0.02). There were no other statistically different results with any of the analytes we tested.

Numerous studies of cervical mucus have used indirect tools such as PCR and RNA for quantitation; however, there are considerable post-synthesis modifications to the basic proteins, thus making these methods indirect as they fail to actually measure what is actually present [5, 11, 26, 35, 63, 76].

We were somewhat surprised that we did not observe more differences when the values were compared between women in the proliferative versus the follicular phases; only MUC1 and MUC4 values were statistically significantly different (*P* = 0.04 MUC1 and MUC4 versus all others).

The most interesting and surprising conclusion from these studies is that the influence of hormones is much less far reaching than that of the microflora. Both gel-forming and secreted mucins increase significantly in women with BV. These observations are consistent with many of the *in vitro* tissue models that demonstrated mucin production is up-regulated by bacterial metabolic products [63, 67, 76]

It was believed that MUC1 was released from the cell surface to allow attachment of the fertilized egg [1, 79, 80] but recently, it was determined that it is MUC16 that prevents cellular adhesion [1, 79–81]. Our results suggest that the three different contraceptives act differently on the control of mucin in the reproductive tract. MUC1 CVF values in the Depo-Provera group are higher than all the other groups except the pre-menopausal (days 1–15), in which we expect MUC1 to be higher [1, 38, 80, 82]. Mirena IUD users present values that differ from the other groups. Of the women using hormonal contraception, the Depo-Provera group demonstrated the highest levels of MUC1 and MUC4.

MUC1 and MUC4 are cell surface associated mucins and MUC5AC and MUC7 are gel- forming mucins; all increased significantly during the transition from normal to intermediate to bacterial vaginosis. The levels of MUC1 protein were three to nine times higher in CVF samples from women with BV.

The strong influence of the microflora on the expression of the mucins suggested that the inclusion of women with BV influences some of the observations of the cohort and when they were stratified by hormonal status (data not shown). Therefore, we analyzed the data excluding women with BV. These are novel observations, but are consistent with other works that reported that MUC5AC and MUC4 transcripts are upregulated *in vitro* by gram-positive and gram-negative organisms in respiratory explants [63]. MUC4 genes are important as they code for a sialoglycoprotein complex in the female genital tract [83, 84]. Increasingly, mucins are recognized as elements of an inflammatory response [85]. MUC5AC, Toll Like Receptors, broad spectrum antimicrobial peptides, IL-6, IL-8 and TNF-α in other systems, are upregulated by lipopolysaccharide and other bacterial components [21, 37, 63, 85–87].

MUC1, MUC4, and MUC5B mRNAs have been studied relative to the menstrual cycle [6, 35] and their role in fertility there has been extensively studied [6, 35, 88, 89]. Because we lacked a reliable antibody, we have not studied the MUC5B mucin. However, MUC5AC appears to behave as it does in other systems, where it responds to inflammatory cytokines and bacterial endotoxins [26, 81, 89, 90], and did not appear to be regulated by methods of contraception or hormonal levels in our study. This suggests a different role for these mucins in the vaginal vault and cervix that is separate from reproduction (unless the mucins play a dual role). The increased quantities of MUC7 observed in women with BV suggest this protein may play a protective role of some sort, as it appears to do in the respiratory system [91].

In this study, we are able to distinguish two distinct sets of phenomena. First, we see the shift of the microflora from the normal flora of primarily gram positive rods and cocci to a BV type of flora dominated by gram negative anaerobic rods. With this shift, we see increases in glycosidase activity, probably of bacterial origin, which is correlated with alterations of the proteins found in the vagina and cervix. We also see how women respond to the shift in their microflora by increasing the levels of specific mucins. These data suggest that MUC5AC and MUC7 function as they do elsewhere in the body, where they are part of the inflammatory response. This is consistent with other observations showing inflammatory cytokines in BV [21, 87, 92].

The distribution of the women by hormonal status in our studies was: post-menopausal (N = 23); pre-menopausal, days 1–14 of cycle (N = 19); pre-menopausal, days 15–30 of cycle (N = 23); those using oral contraceptives (N = 24): DMPA (medroxyprogesterone acetate, Depo-Provera, (N = 19), or the Mirena IUD (N = 25).

We found only one statistically different enzyme result: α-glucosidase activity was lower in postmenopausal women (*P* = <0.001) when compared to all reproductive age women. Because there is a decrease in *Lactobacillus* in postmenopausal women [93–95], we believe the low level of this enzyme activity is a reflection of lower levels of *Lactobacillus* and other microorganisms that secrete the enzyme to utilize poly-glucose polymers like glycogen for carbon and energy.

### Impact on lectin binding

Among the hormonal groups, postmenopausal women had increased SNA binding (detects α-2,6-linked sialic acids) and decreased MAL binding (detects α-2,3-linked sialic acids) bound sialic acid *P* = 0.043 and 0.045, respectively. There were no differences in the content of high mannose structures in any of the hormonal groups as determined by griffithsin binding (Table 1). In a previous study of the cervical-vaginal lavage samples from these same women, the quantities of SNA and MAL bound were comparable to the value we report here. However, in the previous study the quantities of GRFT bound ranged from 5 to 11 pg bound per ng protein, but here we report values ranging from 155 to 217 pg bound per ng protein. Thus, the CVF has about 18–20 fold higher concentrations of GRFT (binding sites) than does the CVL. The changes in sialic acid lectin binding were small and certainly less than we anticipated considering the mRNA work as well as our earlier studies with the CVL samples from the same women [1, 3, 5]. We did observe a major difference in the amount GRFT binding proteins. It is difficult to understand why these values did not change in concert. It at very least suggests there is a substantial loss of one or more of the proteins that have left the cervix to become part of the vaginal fluid and further indicates the extent to which microbial action is occurring. With MAL and SNA and our other lectins it is possible there may be some contribution by bacteria since it is well established that many bacteria display sialic acids on their surfaces [1, 3, 5, 32, 96, 97]. But as of yet high mannose, the GRFT binding structure, has not been identified in bacteria.

### Impact on MUC concentrations

We observed higher, but not highly statistically significant, levels of MUC1 in days 1–14 of cycle and DMPA-using women; MUC4 was lower in days 15–30 of cycle (*P* = 0.02); MUC5AC was lower in postmenopausal women and women using IUDs; and MUC7 was lower in IUD-using women (Table 2).

Overall, when compared with our previous studies of CVLs, the values observed for the enzyme activities were much lower in the CVFs than the CVLs. The quantities of sialic acid binding lectins bound were almost identical in the CVL and CVF samples of postmenopausal women. This suggests that vaginal bacteria are responsible for the enzyme activity, as the concentration of bacteria in the CVLs are about 10^8^ or more and there are very few organisms in or on the cervix of healthy women. Furthermore, it indicates that the cervical mucus samples collected are relatively free of bacteria, or that bacterial enzymes could contaminate the Catamenial cup during sample collection.

## Conclusions

We have demonstrated that in the CVFs of women with bacterial vaginosis, there are greater amounts of four of the six glycosidases studied and we think it highly likely these enzymes are of bacterial origin. These enzymes may be responsible for altering the vaginal and cervical glycomes. CVF sialidase activity is associated with decreases in the binding of sialic acid binding lectins. Women with BV also had significantly less high mannose binding sites as well as increases in all four of the mucins measured, compared to normal or intermediate scoring women. The decrease amount of GRFT binding sites could be an important key in understanding the impact of BV on innate immunity.

When we stratified by hormonal status, we were able to detect a very subtle picture. The post-menopausal women bound more SNA (specific for α-2-6 linked sialic acids) and less MAL (specific for α-2-3 linked sialic acids), but there was no difference in GRFT binding. The mucin levels demonstrated very subtle differences that were affected by hormones. MUC1 was higher in the first 14 days of the menstrual cycle and in women using DMPA. MUC4 was lower in days 15–30 of the cycle. IUD users had lower MUC5AC and MUC7 and post-menopausal women had lower MUC7. Quantitatively, the data presented here are comparable to those present in our work with CVLs, except for the binding of GRFT that is far greater (20–30 fold) in the CVF than the CVL. Thus, the cervix and vaginal vault glycomes are dynamic systems that respond to internal signals such as hormones and to other signals such as the vaginal microbiome.

## Supporting Information

S1 Table Reagents used for the work presented. (DOCX)

S2 Table Primary data used to derive the data presented. (XLSX)

## References

[1] Carson D, DeSouza M, Kardon R, Zhou X, Lagow E, Julian J. Mucin expression and function in the female reproductive tract. Human Reproduction Update. 1998;4(5):459–64. 10.1093/humupd/4.5.459 10027596

[2] Cone RA. Barrier properties of mucus. Adv Drug Deliv Rev. 2009;61(2):75–85. Epub 2009/01/13. 10.1016/j.addr.2008.09.008 . 19135107

[3] Dekker J, Rossen JW, Buller HA, Einerhand AW. The MUC family: an obituary. Trends Biochem Sci. 2002;27(3):126–31. Epub 2002/03/15. . 11893509 10.1016/s0968-0004(01)02052-7

[4] Forstner G. Signal transduction, packaging and secretion of mucins. Annu Rev Physiol. 1995;57:585–605. Epub 1995/01/01. 10.1146/annurev.ph.57.030195.003101 . 7778879

[5] Gipson I, Spurr-Michaud S, Moccia R, Zhan Q, Toribara N, Ho S, et al MUC4 and MUC5B transcripts are the prevalent mucin messenger ribonucleic acids of the human endocervix. Biol Reprod. 1999;60:58–64. 9858486 10.1095/biolreprod60.1.58

[6] Gipson IK, Moccia R, Spurr-Michaud S, Argueso P, Gargiulo AR, Hill JA 3rd, et al The Amount of MUC5B mucin in cervical mucus peaks at midcycle. J Clin Endocrinol Metab. 2001;86(2):594–600. Epub 2001/02/07. . 11158014 10.1210/jcem.86.2.7174

[7] Venkataraman N, Cole AL, Svoboda P, Pohl J, Cole AM. Cationic Polypeptides Are Required for Anti-HIV-1 Activity of Human Vaginal Fluid. The Journal of Immunology. 2005;175(11):7560–7. 16301665 10.4049/jimmunol.175.11.7560

[8] Vogel I, Gronbaek H, Flyvbjerg A, Thorsen P. Albumin in vaginal secretion is a marker of infection. Int J Gynaecol Obstet. 2003;83:307–8. 14643045 10.1016/s0020-7292(03)00268-6

[9] Corfield AP, Wagner SA, Clamp JR, Kriaris MS, Hoskins LC. Mucin degradation in the human colon: production of sialidase, sialate O-acetylesterase, N-acetylneuraminate lyase, arylesterase, and glycosulfatase activities by strains of fecal bacteria. Infection and Immunity. 1992;60(10):3971–8. Epub 1992/10/01. 1398908 10.1128/iai.60.10.3971-3978.1992 PMC257425

[10] Roberton A, Corfield A. Mucin degradation and its significance in inflammatory conditions of the gastrointestinal tract In: Tannock G, editor. Medical Importance of the Normal Microflora: Springer US; 1999 p. 222–61.

[11] Andrianifahanana M, Moniaux N, Batra SK. Regulation of mucin expression: mechanistic aspects and implications for cancer and inflammatory diseases. Biochimica et Biophysica Acta (BBA)-Reviews on Cancer. 2006;1765(2):189–222. 16487661 10.1016/j.bbcan.2006.01.002

[12] Cohen M, Varki A. The sialome—far more than the sum of its parts. Omics: a journal of integrative biology. 2010;14(4):455–64. 10.1089/omi.2009.0148 20726801

[13] Corfield AP. Mucins: A biologically relevant glycan barrier in mucosal protection. Biochimica et Biophysica Acta (BBA)—General Subjects. 2015;1850(1):236–52. 10.1016/j.bbagen.2014.05.003. 24821013

[14] McGuckin MA, Lindén SK, Sutton P, Florin TH. Mucin dynamics and enteric pathogens. Nature Reviews Microbiology. 2011;9(4):265–78. 10.1038/nrmicro2538 21407243

[15] Schauer R. Sialic acids as regulators of molecular and cellular interactions. Curr Opin Struct Biol. 2009;19(5):507–14. Epub 2009/08/25. 10.1016/j.sbi.2009.06.003 . 19699080 PMC7127376

[16] Varki A, Schauer R. Sialic Acids In: Varki A, Cummings RD, Esko JD, Freeze HH, Stanley P, Bertozzi CR, et al, editors. Essentials of Glycobiology. 2nd ed Cold Spring Harbor (NY) 2009. 20301239

[17] Moncla LH, Zhong G, Nelson CW, Dinis JM, Mutschler J, Hughes AL, et al Selective Bottlenecks Shape Evolutionary Pathways Taken during Mammalian Adaptation of a 1918-like Avian Influenza Virus. Cell Host & Microbe. 2016;19(2):169–80. 26867176 10.1016/j.chom.2016.01.011 PMC4761429

[18] Strober W, Hanson LÅ, Sell KW. Recent advances in mucosal immunity: Raven Press New York; 1982.

[19] Keller MJ, Madan RP, Shust G, Carpenter CA, Torres NM, Cho S, et al Changes in the soluble mucosal immune environment during genital herpes outbreaks. J Acquir Immune Defic Syndr. 2012;61(2):194–202. Epub 2012/07/24. 10.1097/QAI.0b013e31826867ae . 22820806 PMC3685489

[20] Olmsted SS, Padgett JL, Yudin AI, Whaley KJ, Moench TR, Cone RA. Diffusion of Macromolecules and Virus-Like Particles in Human Cervical Mucus. Biophysical Journal. 2001;81(4):1930–7. 10.1016/S0006-3495(01)75844-4. 11566767 PMC1301668

[21] Valore EV, Park CH, Igreti SL, Ganz T. Antimicrobial components of vaginal fluid. Am J Obstet Gynecol. 2002;187(3):561–8. 12237628 10.1067/mob.2002.125280

[22] Chappell CA, Rohan LC, Moncla BJ, Wang L, Meyn LA, Bunge K, et al The effects of reproductive hormones on the physical properties of cervicovaginal fluid. Am J Obstet Gynecol. 2014 Epub 2014/03/26. 10.1016/j.ajog.2014.03.041 . 24662718 PMC4149850

[23] Chappell CA, Isaacs CE, Xu W, Meyn LA, Uranker K, Dezzutti CS, et al The effect of menopause on the innate antiviral activity of cervicovaginal lavage. Am J Obstet Gynecol. 2015. 10.1016/j.ajog.2015.03.045 PMC4519412 25818668

[24] Hagen KT. O-Glycosylation and Development In: Endo T, Seeberger PH, Hart GW, Wong C-H, Taniguchi N, editors. Glycoscience: Biology and Medicine: Springer Japan; 2014 p. 1–8.

[25] Wilson IBH, Breton C, Imberty A, Tvaroška I. Molecular Basis for the Biosynthesis of Oligo- and Polysaccharides In: Fraser-Reid B, Tatsuta K, Thiem J, editors. Glycoscience: Springer Berlin Heidelberg; 2008 p. 2265–323.

[26] Gipson I, Ho S, Spurr-Michaud S, Tisdale A, Zhan Q, Torlakovic E, et al Mucin genes expressed by human female reproductive tract epithelia. Biol Reprod. 1997;56:999–1011. 9096884 10.1095/biolreprod56.4.999

[27] Moncla BJ, Chappell CA, Mahal LK, Debo BM, Meyn LA, Hillier SL. Impact of Bacterial Vaginosis, as Assessed by Nugent Criteria and Hormonal Status on Glycosidases and Lectin Binding in Cervicovaginal Lavage Samples. PLoS One. 2015;10(5):e0127091 10.1371/journal.pone.0127091 26011704 PMC4444347

[28] Wang L, Koppolu S, Chappell C, Moncla BJ, Hillier SL, Mahal LK. Studying the Effects of Reproductive Hormones and Bacterial Vaginosis on the Glycome of Lavage Samples from the Cervicovaginal Cavity. PLoS One. 2015;10(5):e0127021 10.1371/journal.pone.0127021 25993513 PMC4439148

[29] Roberton AM, Wiggins R, Horner PJ, Greenwood R, Crowley T, Fernandes A, et al A Novel Bacterial Mucinase, Glycosulfatase, Is Associated with Bacterial Vaginosis. Journal of Clinical Microbiology. 2005;43(11):5504–8. 10.1128/jcm.43.11.5504-5508.2005 16272477 PMC1287821

[30] Varki A. Nothing in glycobiology makes sense, except in the light of evolution. Cell. 2006;126(5):841–5. 16959563 10.1016/j.cell.2006.08.022

[31] Varki NM, Varki A. Diversity in cell surface sialic acid presentations: implications for biology and disease. Lab Invest. 2007;87(9):851–7. 17632542 10.1038/labinvest.3700656 PMC7100186

[32] Hillier SL, Nugent RP, Eschenbach DA, Krohn MA, Gibbs RS, Martin DH, et al Association between bacterial vaginosis and preterm delivery of a low-birth-weight infant. The Vaginal Infections and Prematurity Study Group. N Engl J Med. 1995;333(26):1737–42. Epub 1995/12/28. 10.1056/NEJM199512283332604 . 7491137

[33] Klebanoff MA, Hillier SL, Nugent RP, MacPherson CA, Hauth JC, Carey JC, et al Is bacterial vaginosis a stronger risk factor for preterm birth when it is diagnosed earlier in gestation? Am J Obstet Gynecol. 2005;192(2):470–7. Epub 2005/02/08. 10.1016/j.ajog.2004.07.017 . 15695989

[34] Nugent RP, Krohn MA, Hillier SL. Reliability of diagnosing bacterial vaginosis is improved by a standardized method of gram stain interpretation. Journal of Clinical Microbiology. 1991;29(2):297–301. Epub 1991/02/01. 1706728 10.1128/jcm.29.2.297-301.1991 PMC269757

[35] Gipson IK. Mucins of the human endocervix. Front Biosci. 2001;6:D1245–55. Epub 2001/10/02. . 11578960 10.2741/gipson

[36] Braham PH, Moncla BJ. Rapid presumptive identification and further characterization of Bacteroides forsythus. Journal of Clinical Microbiology. 1992;30(3):649–54. Epub 1992/03/01. 1551981 10.1128/jcm.30.3.649-654.1992 PMC265126

[37] Cauci S, Culhane JF, Di Santolo M, McCollum K. Among pregnant women with bacterial vaginosis, the hydrolytic enzymes sialidase and prolidase are positively associated with interleukin-1beta. Am J Obstet Gynecol. 2008;198(1):132 e1–7. Epub 2007/08/24. 10.1016/j.ajog.2007.05.035 . 17714681

[38] Cauci S, McGregor J, Thorsen P, Grove J, Guaschino S. Combination of vaginal pH with vaginal sialidase and prolidase activities for prediction of low birth weight and preterm birth. Am J Obstet Gynecol. 2005;192(2):489–96. Epub 2005/02/08. 10.1016/j.ajog.2004.07.023 . 15695992

[39] McGregor JA, French JI, Jones W, Milligan K, McKinney PJ, Patterson E, et al Bacterial vaginosis is associated with prematurity and vaginal fluid mucinase and sialidase: results of a controlled trial of topical clindamycin cream. Am J Obstet Gynecol. 1994;170(4):1048–59; discussion 59–60. Epub 1994/04/01. . 8166188 10.1016/s0002-9378(94)70098-2

[40] Moncla BJ, Braham P, Hillier SL. Sialidase (neuraminidase) activity among gram-negative anaerobic and capnophilic bacteria. Journal of Clinical Microbiology. 1990;28(3):422–5. Epub 1990/03/01. 2108991 10.1128/jcm.28.3.422-425.1990 PMC269635

[41] Moncla BJ, Braham P, Rabe LK, Hillier SL. Rapid presumptive identification of black-pigmented gram-negative anaerobic bacteria by using 4-methylumbelliferone derivatives. Journal of Clinical Microbiology. 1991;29(9):1955–8. Epub 1991/09/01. 1774320 10.1128/jcm.29.9.1955-1958.1991 PMC270241

[42] Moncla BJ, Pryke KM. Oleate lipase activity in Gardnerella vaginalis and reconsideration of existing biotype schemes. BMC Microbiol. 2009;9:78 Epub 2009/04/24. 10.1186/1471-2180-9-78 19386125 PMC2680412

[43] Roberts G, Tarelli E, Homer KA, Philpott-Howard J, Beighton D. Production of an Endo-β-N-Acetylglucosaminidase Activity Mediates Growth of Enterococcus faecalis on a High-Mannose-Type Glycoprotein. Journal of Bacteriology. 2000;182(4):882–90. PMC94360. 10648510 10.1128/jb.182.4.882-890.2000 PMC94360

[44] Birse KM, Burgener A, Westmacott GR, McCorrister S, Novak RM, Ball TB. Unbiased Proteomics Analysis Demonstrates Significant Variability in Mucosal Immune Factor Expression Depending on the Site and Method of Collection. PLoS One. 2013;8(11):e79505 10.1371/journal.pone.0079505 24244515 PMC3828359

[45] Fields S, Song B, Rasoul B, Fong J, Works MG, Shew K, et al New Candidate Biomarkers in the Female Genital Tract to Evaluate Microbicide Toxicity. PLoS One. 2014;9(10):e110980 10.1371/journal.pone.0110980 25333937 PMC4205019

[46] Mitchell C, Paul K, Agnew K, Gaussman R, Coombs RW, Hitti J. Estimating volume of cervicovaginal secretions in cervicovaginal lavage fluid collected for measurement of genital HIV-1 RNA levels in women. Journal of clinical microbiology. 2011;49(2):735–6. 10.1128/JCM.00991-10 21106793 PMC3043490

[47] Lowry OH, Rosebrough NJ, Farr AL, Randall RJ. Protein measurement with the Folin phenol reagent. J Biol Chem. 1951;193(1):265–75. 14907713

[48] Moncla B, Charnetzky W. Free form lipoproteins in Yersinia pestis. Microbios letters. 1986;32(126):81–6.

[49] Patton DL, Cosgrove Sweeney YT, Balkus JE, Rohan LC, Moncla BJ, Parniak AA, et al Preclinical safety assessments of UC781 anti-human immunodeficiency virus topical microbicide formulations. Antimicrobial Agents and Chemotherapy. 2007;51(5):1608–15. 10.1128/aac.00984-06 WOS:000246541500003. 17353240 PMC1855550

[50] Lewis WG, Robinson LS, Gilbert NM, Perry JC, Lewis AL. Degradation, foraging, and depletion of mucus sialoglycans by the vagina-adapted Actinobacterium Gardnerella vaginalis. J Biol Chem. 2013;288(17):12067–79. Epub 2013/03/13. 10.1074/jbc.M113.453654 23479734 PMC3636892

[51] Lewis WG, Robinson LS, Perry J, Bick JL, Peipert JF, Allsworth JE, et al Hydrolysis of secreted sialoglycoprotein immunoglobulin A (IgA) in ex vivo and biochemical models of bacterial vaginosis. J Biol Chem. 2012;287(3):2079–89. Epub 2011/12/03. 10.1074/jbc.M111.278135 22134918 PMC3265887

[52] Briselden AM, Moncla BJ, Stevens CE, Hillier SL. Sialidases (neuraminidases) in bacterial vaginosis and bacterial vaginosis-associated microflora. Journal of Clinical Microbiology. 1992;30(3):663–6. 1551983 10.1128/jcm.30.3.663-666.1992 PMC265128

[53] Howe L, Wiggins R, Soothill PW, Millar MR, Horner PJ, Corfield AP. Mucinase and sialidase activity of the vaginal microflora: implications for the pathogenesis of preterm labour. Int J STD AIDS. 1999;10(7):442–7. Epub 1999/08/24. . 10454178 10.1258/0956462991914438

[54] Lopes dos Santos Santiago G, Deschaght P, El Aila N, Kiama TN, Verstraelen H, Jefferson KK, et al Gardnerella vaginalis comprises three distinct genotypes of which only two produce sialidase. Am J Obstet Gynecol. 2011;204(5):450.e1–.e7. 10.1016/j.ajog.2010.12.061. 21444061

[55] Homer KA, Whiley RA, Beighton D. Production of specific glycosidase activities by Streptococcus intermedius strain UNS35 grown in the presence of mucin. Journal of Medical Microbiology. 1994;41(3):184–90. 10.1099/00222615-41-3-184 8064838

[56] King SJ, Hippe KR, Weiser JN. Deglycosylation of human glycoconjugates by the sequential activities of exoglycosidases expressed by Streptococcus pneumoniae. Mol Microbiol. 2006;59(3):961–74. 16420364 10.1111/j.1365-2958.2005.04984.x

[57] Wiggins R, Hicks SJ, Soothill PW, Millar MR, Corfield AP. Mucinases and sialidases: their role in the pathogenesis of sexually transmitted infections in the female genital tract. Sex Transm Infect. 2001;77(6):402–8. 10.1136/sti.77.6.402 11714935 PMC1744407

[58] Homer KA, Roberts G, Byers HL, Tarelli E, Whiley RA, Philpott-Howard J, et al Mannosidase production by viridans group streptococci. Journal of clinical microbiology. 2001;39(3):995–1001. Epub 2001/03/07. 10.1128/JCM.39.3.995-1001.2001 11230417 PMC87863

[59] King SJ. Pneumococcal modification of host sugars: a major contributor to colonization of the human airway? Molecular Oral Microbiology. 2010;25(1):15–24. 10.1111/j.2041-1014.2009.00564.x 20331791

[60] Roberts G, Homer KA, Tarreti E, Philpott-Howard J, Devriese LA, Beighton D. Distribution of endo-β-N-acetylglucosaminidase amongst enterococci. Journal of Medical Microbiology. 2001;50(7):620–6. 11444772 10.1099/0022-1317-50-7-620

[61] Hart ML, Saifuddin M, Uemura K, Bremer EG, Hooker B, Kawasaki T, et al High mannose glycans and sialic acid on gp120 regulate binding of mannose-binding lectin (MBL) to HIV type 1. AIDS Res Hum Retroviruses. 2002;18(17):1311–7. 12487819 10.1089/088922202320886352

[62] Ji X, Gewurz H, Spear GT. Mannose binding lectin (MBL) and HIV. Molecular immunology. 2005;42(2):145–52. 15488604 10.1016/j.molimm.2004.06.015

[63] Dohrman A, Miyata S, Gallup M, Li JD, Chapelin C, Coste A, et al Mucin gene (MUC 2 and MUC 5AC) upregulation by Gram-positive and Gram-negative bacteria. Biochim Biophys Acta. 1998;1406(3):251–9. Epub 1998/06/19. . 9630659 10.1016/s0925-4439(98)00010-6

[64] Radtke AL, Quayle AJ, Herbst-Kralovetz MM. Microbial products alter the expression of membrane-associated mucin and antimicrobial peptides in a three-dimensional human endocervical epithelial cell model. Biology of Reproduction. 2012;87(6):132 Epub 2012/10/12. 10.1095/biolreprod.112.103366 . 23053434 PMC4435425

[65] Lemjabbar H, Basbaum C. Platelet-activating factor receptor and ADAM10 mediate responses to Staphylococcus aureus in epithelial cells. Nat Med. 2002;8(1):41–6. 11786905 10.1038/nm0102-41

[66] Linden SK, Sutton P, Karlsson NG, Korolik V, McGuckin MA. Mucins in the mucosal barrier to infection. Mucosal Immunol. 2008;1(3):183–97. 10.1038/mi.2008.5 19079178 PMC7100821

[67] Smirnova MG, Guo L, Birchall JP, Pearson JP. LPS up-regulates mucin and cytokine mRNA expression and stimulates mucin and cytokine secretion in goblet cells. Cellular Immunology. 2003;221(1):42–9. 10.1016/S0008-8749(03)00059-5. 12742381

[68] Yanagihara K, Seki M, Cheng P-W. Lipopolysaccharide induces mucus cell metaplasia in mouse lung. American journal of respiratory cell and molecular biology. 2001;24(1):66–73. 11152652 10.1165/ajrcmb.24.1.4122

[69] Appelmelk BJ, Monteiro MA, Martin SL, Moran AP, Vandenbroucke-Grauls CM. Why Helicobacter pylori has Lewis antigens. Trends in microbiology. 2000;8(12):565–70. 11115753 10.1016/s0966-842x(00)01875-8

[70] Gallego MP, Hulen C. Influence of sialic acid and bacterial sialidase on differential adhesion of Pseudomonas aeruginosa to epithelial cells. Colloids and Surfaces B: Biointerfaces. 2006;52(2):154–6. 16781124 10.1016/j.colsurfb.2006.04.013

[71] Pang SS, Nguyen STS, Perry AJ, Day CJ, Panjikar S, Tiralongo J, et al The Three-dimensional Structure of the Extracellular Adhesion Domain of the Sialic Acid-binding Adhesin SabA from Helicobacter pylori. Journal of Biological Chemistry. 2014;289(10):6332–40. 10.1074/jbc.M113.513135 24375407 PMC3945300

[72] Biswas N, Rodriguez-Garcia M, Shen Z, Crist SG, Bodwell JE, Fahey JV, et al Effects of Tenofovir on Cytokines and Nucleotidases in HIV-1 Target Cells and the Mucosal Tissue Environment in the Female Reproductive Tract. Antimicrobial Agents and Chemotherapy. 2014;58(11):6444–53. 10.1128/aac.03270-14 25136003 PMC4249387

[73] Lehman DA, Ronen K, Blish CA, Baeten JM, Jalalian-Lechak Z, Jaoko W, et al Systemic Cytokine Levels Show Limited Correlation With Risk of HIV-1 Acquisition. JAIDS Journal of Acquired Immune Deficiency Syndromes. 2014;66(2):135–9 10.1097/QAI.0000000000000104 24413043 PMC4020985

[74] Mueller Y, Fraietta J, Ratner D, Boesteanu A, Gupta P, Katsikis P. Anti-HIV, anti-inflammatory and safety profile of the PDB microbicide candidate (HUM8P.342). The Journal of Immunology. 2014;192(1 Supplement):185 17.

[75] Stute P, Kollmann Z, Bersinger N, von Wolff M, Thurman AR, Archer DF. Vaginal cytokines do not differ between postmenopausal women with and without symptoms of vulvovaginal irritation. Menopause. 2014;21(8):1. 24398407 10.1097/GME.0000000000000179

[76] Li X, Wang L, Nunes DP, Troxler RF, Offner GD. Pro-inflammatory Cytokines Up-regulate MUC1 Gene Expression in Oral Epithelial Cells. Journal of Dental Research. 2003;82(11):883–7. 10.1177/154405910308201107 14578499

[77] Albertsmeyer A-C, Kakkassery V, Spurr-Michaud S, Beeks O, Gipson IK. Effect of pro-inflammatory mediators on membrane-associated mucins expressed by human ocular surface epithelial cells. Exp Eye Res. 2010;90(3):444–51. 10.1016/j.exer.2009.12.009. 10.1016/j.exer.2009.12.009 20036239 PMC2880853

[78] Corti S, Nizzardo M, Nardini M, Donadoni C, Salani S, Ronchi D, et al Embryonic stem cell-derived neural stem cells improve spinal muscular atrophy phenotype in mice. Brain. 2010;133(Pt 2):465–81. Epub 2009/12/25. 10.1093/brain/awp318 . 20032086

[79] Aplin J, Hey N, Li T. MUC1 as a cell surface and secretory component of endometrial epithelium: reduced levels in recurrent miscarriage. Am J Reprod Immunol. 1996;35:261–6. 8962658 10.1111/j.1600-0897.1996.tb00042.x

[80] Brayman M, Thathiah A, Carson Daniel D. MUC1: A multifunctional cell surface component of reproductive tissue epithelia. Reproductive Biology and Endocrinology. 2004;2(1):4. 14711375 10.1186/1477-7827-2-4 PMC320498

[81] Gipson IK, Blalock T, Tisdale A, Spurr-Michaud S, Allcorn S, Stavreus-Evers A, et al MUC16 is lost from the uterodome (pinopode) surface of the receptive human endometrium: in vitro evidence that MUC16 is a barrier to trophoblast adherence. Biology of Reproduction. 2008;78(1):134–42. Epub 2007/10/19. 10.1095/biolreprod.106.058347 . 17942799

[82] Cauci S, Driussi S, Monte R, Lanzafame P, Pitzus E, Quadrifoglio F. Immunoglobulin A response against Gardnerella vaginalis hemolysin and sialidase activity in bacterial vaginosis. Am J Obstet Gynecol. 1998;178(3):511–5. Epub 1998/04/16. . 9539518 10.1016/s0002-9378(98)70430-2

[83] Carraway K, Idris N. Regulation of sialomucin complex/Muc4 in the female rat reproductive tract. Biochem Soc Trans. 2001;29(2):162–6. 11356146 10.1042/0300-5127:0290162

[84] Rossi EA, McNeer RR, Price-Schiavi SA, Van den Brande JMH, Komatsu M, Thompson JF, et al Sialomucin Complex, a Heterodimeric Glycoprotein Complex: EXPRESSION AS A SOLUBLE, SECRETABLE FORM IN LACTATING MAMMARY GLAND AND COLON. Journal of Biological Chemistry. 1996;271(52):33476–85. 10.1074/jbc.271.52.33476 8969211

[85] Zen Y, Harada K, Sasaki M, Tsuneyama K, Katayanagi K, Yamamoto Y, et al Lipopolysaccharide Induces Overexpression of MUC2 and MUC5AC in Cultured Biliary Epithelial Cells: Possible Key Phenomenon of Hepatolithiasis. The American Journal of Pathology. 2002;161(4):1475–84. 10.1016/S0002-9440(10)64423-9. 12368220 PMC1867307

[86] Valore EV, Wiley DJ, Ganz T. Reversible deficiency of antimicrobial polypeptides in bacterial vaginosis. Infection and Immunity. 2006;74(10):5693–702. 16988245 10.1128/IAI.00524-06 PMC1594936

[87] Yudin MH, Landers DV, Meyn L, Hillier SL. Clinical and Cervical Cytokine Response to Treatment With Oral or Vaginal Metronidazole for Bacterial Vaginosis During Pregnancy: A Randomized Trial. Obstetrics & Gynecology. 2003;102(3):527–34. 12962937 10.1016/s0029-7844(03)00566-0

[88] Aplin JD. MUC-1 glycosylation in endometrium: possible roles of the apical glycocalyx at implantation. Human Reproduction. 1999;14(suppl 2):17–25. 10.1093/humrep/14.suppl_2.17 10690797

[89] Escande F, Aubert JP, Porchet N, Buisine MP. Human mucin gene MUC5AC: organization of its 5'-region and central repetitive region. Biochem J. 2001;358(Pt 3):763–72. Epub 2001/09/06. 11535137 10.1042/0264-6021:3580763 PMC1222110

[90] Yurewicz E, Matsuura F, Moghissi K. Structural characterization of neutral oligosaccharides of human midcycle cervical mucin. Journal of Biological Chemistry. 1982;257(5):2314–22. 6277894

[91] Rousseau K, Vinall LE, Butterworth SL, Hardy RJ, Holloway J, Wadsworth MEJ, et al MUC7 Haplotype Analysis: Results from a Longitudinal Birth Cohort Support Protective Effect of the MUC7*5 Allele on Respiratory Function. Annals of Human Genetics. 2006;70(4):417–27. 10.1111/j.1469-1809.2006.00250.x 16759176

[92] Imseis HM, Greig PC, Livengood CH, Shunior E, Durda P, Erikson M. Characterization of the Inflammatory Cytokines in the Vagina During Pregnancy and Labor and With Bacterial Vaginosis. Journal of the Society for Gynecologic Investigation. 1997;4(2):90–4. 10.1177/107155769700400208 9101468

[93] Cauci S, Driussi S, De Santo D, Penacchioni P, Iannicelli T, Lanzafame P, et al Prevalence of bacterial vaginosis and vaginal flora changes in peri-and postmenopausal women. Journal of clinical microbiology. 2002;40(6):2147–52. 12037079 10.1128/JCM.40.6.2147-2152.2002 PMC130764

[94] Jacobson JC, Turok DK, Dermish AI, Nygaard IE, Settles ML. Vaginal microbiome changes with levonorgestrel intrauterine system placement. Contraception. 2014;90(2):130–5. 10.1016/j.contraception.2014.04.006 WOS:000339367700005. 24835828

[95] Petricevic L, Domig KJ, Nierscher FJ, Sandhofer MJ, Krondorfer I, Kneifel W, et al Differences in the vaginal lactobacilli of postmenopausal women and influence of rectal lactobacilli. Climacteric. 2013;16(3):356–61. 10.3109/13697137.2012.725788 WOS:000318937600007. 23113473

[96] Jones AH, Lee CC, Moncla BJ, Robinovitch MR, Birdsell DC. Surface localization of sialic acid on Actinomyces viscosus. Journal of General Microbiology. 1986;132(12):3381–91. Epub 1986/12/01. . 3655719 10.1099/00221287-132-12-3381

[97] Leite B, Hillier S, Moncla BJ. Detection and Localisation of Sialic Acid in Gardnerella vaginalis, Mobiluncus spp., and Other Vaginal Microorganisms. Microbial Ecology in Health and Disease. 1989;2(4):255–60. 10.3109/08910608909140228

