# Supplementary material for: The Effects of Hormones and Vaginal Microflora on the Glycome of the Female Genital Tract: Cervical-Vaginal Fluid
Source: PLoS One. 2016 Jul 20;11(7):e0158687. doi: 10.1371/journal.pone.0158687 (PMC4954690; doi:10.1371/journal.pone.0158687)
Supplement: S1 Table — (DOCX) [file pone.0158687.s001.docx]

**-S1 Table Reagents used for the work presented.**

|  | **Conjugation** | **primary/secondary** | |
| --- | --- | --- | --- |
|  |  |  |  |
| **Ab description / catalogue number** |  |  | **Lot Number** |
|  |  |  |  |
| Anti-MUC1 antibody [HMFG1 (aka 1.10.F3)] | none | primary | GR156588-3a |
| (ab70475) |  |  |  |
|  |  |  |  |
| Anti-MUC4 antibody (ab60720) | none | primary | GR84514 |
|  |  |  |  |
| Anti-MUC7 antibody (ab55542) | none | primary | GR176272-1 |
|  |  |  |  |
| Anti-Mucin 5AC antibody [1-13M1] (ab24070) | none | primary | GR77955 |
|  |  |  |  |
| MUC7 Antibody 0.05 ml (H00004589-A01) | GST tag | primary | 07065-8C11b |
| Mouse Anti-human serum albumin  (ab 10241) | none | primary | GR48379-4 |
| Donkey Anti-Goat IgG H&L (HRP) | HRP | secondary | GR14922-6 |
| preadsorbed (ab97120) |  |  |  |
|  |  |  |  |
| Donkey Anti-Rabbit IgG H&L (HRP) (ab97064) | HRP | secondary | GR6560-7 |
|  |  |  |  |
| Goat Anti-Human IgG Fc (HRP) (ab97225) | HRP | secondary | GR68321-4 |
|  |  |  |  |
| Goat Anti-Mouse IgG H&L (HRP) pre- | HRP | secondary | GR129315-6 |
| adsorbed (ab97040) |  |  |  |
|  |  |  |  |
| Rabbit Anti-Goat IgG H&L (HRP) pre- | HRP | secondary | GR33627-1 |
| adsorbed (ab97105) |  |  |  |
|  |  |  |  |
| Rabbit Anti-Mouse IgG H&L (HRP) (ab6728) | HRP | secondary | GR8317 |
|  |  |  |  |
| Rabbit Anti-Mouse IgG H&L (HRP) (ab97046) | HRP | secondary | GR87741 |
|  |  |  |  |
| Maakia amurensis lectin type II (B-1265) |  | Biotinylated | X0424 |
|  |  |  |  |
| Sambucus nigra lectin (B-1305) |  | Biotinylated | X0611 |
|  |  |  |  |
| Streptavidin (kSA-5014) |  |  | 7A0225 |
|  |  |  |  |

**Griffithsin and anti-Griffithsin antibodies were a generous gift of Kenneth E. Palmer, University of Louisville, Louisville, KY, United States. Human serum albumin was from Sigma-Aldrich St. Louis product number A3782, Lot number 090M700IV.**

**^a^GR lot numbers are products obtained from Abcam, MUC 7 antibody was from Novis and the lectins were obtained from Vector Laboratories, Streptavidin was from Vector Laboratories.**
